# Autonomous Distributed Self-Organization for Mobile Wireless Sensor Networks

**DOI:** 10.3390/s91108961

**Published:** 2009-11-11

**Authors:** Chih-Yu Wen, Hung-Kai Tang

**Affiliations:** Department of Electrical Engineering, Graduate Institute of Communication Engineering, National Chung Hsing University, Taichung 402, Taiwan; E-Mail: g9593005@mail.nchu.edu.tw

**Keywords:** mobile wireless sensor networks, self-organization, topology control

## Abstract

This paper presents an adaptive combined-metrics-based clustering scheme for mobile wireless sensor networks, which manages the mobile sensors by utilizing the hierarchical network structure and allocates network resources efficiently A local criteria is used to help mobile sensors form a new cluster or join a current cluster. The messages transmitted during hierarchical clustering are applied to choose distributed gateways such that communication for adjacent clusters and distributed topology control can be achieved. In order to balance the load among clusters and govern the topology change, a cluster reformation scheme using localized criterions is implemented. The proposed scheme is simulated and analyzed to abstract the network behaviors in a number of settings. The experimental results show that the proposed algorithm provides efficient network topology management and achieves high scalability in mobile sensor networks.

## 1. Introduction

Without a robust infrastructure, sensors in an ad-hoc network may be required to self-organize. Such sensor networks are self-configuring distributed systems and, for reliability, should also operate without centralized control. It has been shown that cluster architecture guarantees basic performance achievement in a mobile ad-hoc network [1, 2]. This effective topology control technique may be advantageous since it (1) conserves limited energy resources and improves energy efficiency, (2) facilitates the spatial reuse of resources to increase the system capacity [3], (3) can construct a virtual backbone for abstracting the characteristics of network topology [4, 5], and (4) makes an ad-hoc network more stable and further provides scalability and robustness for the network [6, 7].

In order to provide reliable communication in wireless ad-hoc networks, maintaining network connectivity is crucial. An implementation of the linked cluster architecture may consider the following tasks: cluster formation, cluster connectivity, and cluster reorganization. This paper presents an adaptive combined-metrics-based clustering scheme for mobile sensor networks, which manages the mobile sensors by utilizing the hierarchical network structure and allocates network resources efficiently. In order to not to rely on a central controller, clustering is carried out by adaptive distributed control techniques via random waiting timers, which takes a number of metrics into account for cluster configuration, including neighboring node properties, residual energy level, and node mobility. To this end, the *Self-Adaptive Mobile Clustering Algorithm* (SAMCA) forms clusters and links in two phases: (I) cluster formation and (II) cluster maintenance.

In Phase I, clusterheads are selected and cluster members are assigned. The proposed algorithm generalizes the previous work [8] to include the motion by using the velocities of the sensors and the characteristics of the network in the waiting timers such that the mobile sensors can be organized automatically in an ad-hoc network. Each sensor operates independently, monitoring communication among its neighbors. Based on the number of neighbors, the node mobility, and a randomized timer, each sensor either joins a nearby cluster, or else forms a new cluster with itself as clusterhead. Established upon bidirectional message exchanges and the cluster architecture, sensors are selected as gateways for adjacent clusters in a fully distributed way.

Once the network topology is specified (as a hierarchical collection of clusters and distributed gateways), maintenance of the linked cluster architecture becomes an issue. In order to govern the hierarchical structure and the topology change efficiently, in Phase II a cluster reformation scheme using localized criterions is applied, especially on merger and division of clusters and reselection of clusterhead and gateway.

As we know, the clustering protocol is vital for a network to achieve scalability. However, a cluster-based network infrastructure requires extra cost for constructing and maintaining a cluster structure compared with a flat-based one. Therefore, the cost of clustering is a key issue to validate the effectiveness and scalability enhancement of a cluster structure. This paper explores the cost of the proposed scheme such as energy consumption for cluster formation and maintenance, communication complexity, and time complexity. By analyzing the proposed clustering scheme in different aspects qualitatively or quantitatively such as neighboring node properties, node density change, and simplified models for describing the process of cluster formation, its robustness and scalability can be clearly specified. The analytical results are compared to the behavior of the algorithm in a number of settings.

The rest of the paper is organized as follows: Section 2. reviews the current literature on clustering techniques for mobile ad-hoc networks. Section 3. describes the procedures of the SAMCA method. Section 4. presents the performance analysis of the SAMCA. In this section, the behavior of the proposed protocol is abstracted using neighboring sensor properties and simplified models, which serve to approximate its performance. Section 5. examines the energy usage of the algorithm. The result provided in [9] is used to investigate situations where the minimum transmission range ensures that the network has a reasonable connectivity. Section 6. verifies the approximation of the desired global behavior and examine the contributions of node mobility to the performance of the proposed scheme. The final section draws concludes of the proposed clustering technique.

## 2. Related Work

Cluster-based self-organization is an important research topic for mobile ad-hoc networks since clustering allows to provide basic levels of system performance. A large variety of approaches for ad-hoc clustering have been proposed in literature [10–45]. Most of these design approaches are heuristic protocols in which each sensor must maintain knowledge of the complete network or identify a subset of sensors with a clusterhead to partition the network into clusters in heuristic ways. Perhaps the earliest of the clustering methods is the identifier-based heuristic called the Linked Cluster Algorithm (LCA) [13], which elects sensor to be a clusterhead if the sensor has the highest identification number among all sensors within one hop of its neighbors. The connectivity-based heuristic of [14, 15] selects the sensors with the maximum number of 1-hop neighbors (i.e., highest degree) to be clusterheads. However, These algorithms suffer from dynamic network topology, which triggers frequent changes of clusterheads. The Max-Min D-cluster Algorithm [16] generates *d*-hop clusters with a complexity of *O*(*d*) without time synchronization. It provides load balancing among clusterheads in the network. Simulation results suggest that this heuristic is superior to the LCA and connectivity-based solutions.

The Low-Energy Adaptive Clustering Hierarchy (LEACH) of [28] utilizes randomized rotation of clusterheads to balance the energy load among the sensors and uses localized coordination to enable scalability and robustness for cluster set-up and operation. LEACH-C (Centralized) [29] uses a centralized controller. The main drawbacks of this algorithm are nonautomatic clusterhead selection and the requirement that the position of all sensors must be known. LEACH's stochastic algorithm is extended in [30] with a deterministic clusterhead selection. Simulation results demonstrate that an increase of network lifetime can be achieved compared with the original LEACH protocol. In [31], the clustering is driven by minimizing the energy spent in wireless sensor networks. The authors adopt the energy model in [28] and use the Subtractive Clustering Algorithm and Fuzzy C-mean (FCM) algorithm to form clusters. Although the above algorithms carefully consider the energy required for clustering, they are not extensively analyzed (due to their complexity) and there is no way of estimating how many clusters will form in a given network.

In [32], a mobility-aware clustering algorithm (MOBIC) is proposed. MOBIC presents an aggregate local mobility metric for the cluster formation process such that mobile nodes with low speed relative to their neighbors have the chance to become clusterheads. However, it neglects mobility manners in later cluster maintenance, which may not work well in a dynamic environment. The Weighted Clustering Algorithm (WCA) [33] considers the number of neighbors, transmission power, mobility, and battery usage in choosing clusters. It limits the number of sensors in a cluster so that clusterheads can handle the load without degradation in performance. These clustering methods rely on synchronous clocking for the exchange of information among sensors which typically limits these algorithms to smaller networks [34]. The authors in [35] characterize the different node parameters in terms of an information theoretic metric and use entropy as a measure of local and mutual information available to every node, such as mobility, energy, and degree in the clusterhead selection procedure. The results demonstrate that the mutual information captured through entropy is very effective in determining the most suitable clusterheads. Basagni *et al.* [36] consider generic metrics (e.g., protocol duration, energy consumption, message overhead, route length, and backbone size) to generate ad-hoc clustering and backbone formation. The node with the highest weight among its neighbors is chosen as a cluster-head. Note that all of the above algorithms generate one-hop clusters, which may be suitable only for small networks.

The authors of [16] generalize the clustering heuristics such that an ordinary node can be at most *d* hops away from its clusterhead, which allows more control and flexibility in the determination of clusterhead density. However, clusters are formed heuristically without taking node mobility and their mobility pattern into consideration. [37] designs a clustering algorithm (DDCA) that adaptively changes its clustering criteria based on the current node mobility. As a multi-hop clustering, DDCA normally forms clusters with larger size compared with 1-hop clusters, which may result in increased control overhead for cluster maintenance. In [38], an adaptive clustering protocol is proposed to maintain a network structure based on load-balancing clustering. In order to reorganize the cluster, the proposed scheme sets upper and lower limits on the cluster size. However, it does not describe the procedures of cluster formation and how to select proper values for the upper and lower bounds of the cluster parameters.

Reference [3] describes a self-organizing, multihop, mobile radio network which relies on a code-division access scheme for multimedia support. A distributed algorithm with a limited number of hops in the cluster is applied to control the network topology. However, this algorithm does not put any restriction on clusters size, which may increase the difficulty of cluster maintenance and lead to performance degradation in dense networks. Nocetti *et al.* [39] study node connectivity and node ID as two particular weights to generalize the cluster definition and formation algorithm so that a cluster contains all nodes that are at distance at most *k* hops from the clusterhead (for *k* = 1 and *k* = 2). Nevertheless, this proposed scheme forms 1-hop and 2-hop clusters without taking into account the cluster size. *K*-clustering frameworks [40, 41] are proposed to divide the wireless network into non-overlapping clusters, and where every two wireless nodes in a cluster are at most *k* hops from each other. Thus, the cluster structure is controlled by hop distance. Although these algorithms have polynomial time and message complexity, unbalanced clusters may be formed due to the lack of information regarding cluster diameter and cluster size.

LCC [42] is designed to minimize clusterhead changes. However, with the increasing node speed, the migration of the cluster members towards others clusters and the role reassignment between two neighboring clusterheads may lead to frequent clusterhead changes. Moreover, a ripple effect may be generated due to the propagation of clusterhead changes in the LCC scheme. In the purpose to generate balanced clusters, [43] restricts both diameter and size of the cluster. Two thresholds are used to control the size of generated clusters and the distances between cluster members and their corresponding clusterhead are at most two hops. However, the logic behind the setting of the two thresholds and the process of achieving inter-cluster communication are not provided. Comprehensive surveys of recently proposed clustering algorithms for mobile ad-hoc and sensor networks can be found in [44, 45].

In our previous works, the sensors are assumed to be fixed and the cluster size is not specified for cluster formation [8]. Moreover, we focus on only the procedures of clusterhead re-selection [46], not the operations of cluster maintenance and distributed gateway selection. In order to manage cluster members and minimize the overhead of intra-cluster communications in a mobile network, the appropriate design of cluster size and the procedures for cluster maintenance are crucial. Therefore, the proposed self-organization strategy (SAMCA) investigates the design of cluster size and considers several important factors such as the contribution of sensor mobility, a sensor joining/leaving a cluster, the operation of cluster merging/splitting, and clusterhead/gateway reselection, in order to balance the load for each cluster and to keep the hierarchical structure efficient. It applies localized criterions and maintains clusters in a fully distributed way. The comparison of the proposed scheme and other cluster-based approaches [3, 33, 41, 43] is further discussed in Section 6..

## 3. The Self-Adaptive Mobile Clustering Algorithm (SAMCA)

This section describes a dynamic distributed algorithm for organizing the mobile sensors using a timer and the characteristics of a network. The main assumptions are: (1) All sensors are homogeneous with the same transmission range; (2) Symmetric communication channel: all links between sensors are bidirectional; (3) Each sensor periodically broadcasts its local information to its neighbors. Note that there are no base stations to coordinate or supervise activities among sensors.

### 3.1. Phase I: Cluster Formation

#### Clusterhead Selection

In order to transit network operation from transitional phase to steady phase smoothly, the system warm-up time may be required in a mobile network. The study in [47] suggests that the warm-up time is a function of node velocity, transmission range, network field, and the mobility model. Assume the value of warm-up time of sensor *i*, *WU_i_*, is a sample from the distribution *C*+*λ*·*U*(0, 1), where *C* and *λ* are positive numbers, the use of parameter *λ* is used to specify the sampling range of the warm-up time, and *U*(0, 1) is a uniform distribution. In our case, the positive number *C* may be considered as an important indicator to capture the warm-up process of the SAMCA protocol (detailed in Section 6.7.).

**Figure 1.**
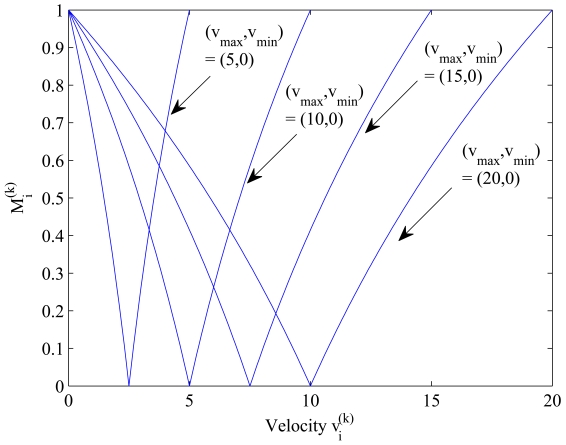
The update strategy for the random waiting time of sensor *i* with varying velocity.

After passing the warm-up time, each sensor sets a random waiting timer for selecting clusterheads. Similar to the setting of warm-up time, the initial value of waiting time of sensor *i*, *WT_i_*, may be a sample from the distribution *C*+*λ*·*U*(0, 1). Each sensor broadcasts a 1-hop *Hello* message and listens for its neighbor's *Hello*, which allows each sensor to estimate how many neighbors it has. Then sensors update their neighbor information and decrease the random waiting time based on the node mobility and each ‘new’ *Hello* message received. To avoid quickly losing the connectivity with neighboring sensors after being a clusterhead, the node mobility of a clusterhead may not be high or low such that the frequency of structure changes may be suppressed. Therefore, the sensors with many neighbors and modest mobility may be good candidates for initiating new clusters; those with few neighbors and high or low mobility may choose to wait. One strategy for updating the random waiting time of sensor *i* is

(1) $$WT_{i}^{\left(k+1\right)}={\text{log}}_{2}\;\left(1+\frac{\left|\upsilon_{i}^{\left(k\right)}-\frac{1}{2}\left(\upsilon_{\max}+\upsilon_{\min}\right)\right|}{\frac{1}{2}\left(\upsilon_{\max}-\upsilon_{\min}\right)}\right)\cdot \rho \cdot WT_{i}^{\left(k\right)}=M_{i}^{\left(k\right)}\cdot \rho \cdot WT_{i}^{\left(k\right)},$$

where $WT_{i}^{\left(k\right)}$ is the waiting time of sensor *i* at time step *k* and 0 < *ρ* < 1, $\upsilon_{i}^{\left(k\right)}$ is the speed of sensor *i* at time step *k* and *υ*_max_ and *υ*_min_ are known bounds on the velocity with 0 ≤ *υ*_min_ < *υ*_max_, and $M_{i}^{\left(k\right)}$ represents the log_2_(·) function. As shown in Figure 1, the log_2_(·) function is with maximum value 1 as $\upsilon_{i}^{\left(k\right)}=\upsilon_{\max}$ or *υ*_min_ and minimum value 0 as $\upsilon_{i}^{\left(k\right)}=\frac{1}{2}\left(\upsilon_{\max}+\upsilon_{\min}\right)$. Note that the proposed mechanism, considering the neighboring density and node mobility, suggests a sensible approach for adjusting the waiting time.

If the random waiting timer expires (i.e., *WT_i_* = 0) and none of the neighboring sensors are already members of a cluster, then sensor *i* declares itself a clusterhead and broadcasts a 1-hop message proclaiming that it is beginning a new cluster; this also serves to notify its neighbors that they are assigned to join the new cluster with ID *i*. When a sensor joins the cluster, it sends an updated *Hello* message and stops its waiting timer. In order to balance the load among clusters, the appropriate setting for the desired cluster size in a mobile network is crucial. Therefore, a reference cluster size *z* is defined to maintain network operation. Accordingly, a clusterhead may choose its cluster members using the distance information (e.g. selecting the nearest neighboring sensors) based on this reference cluster size *z* (described in Section 4.1.).

**Figure 2.**
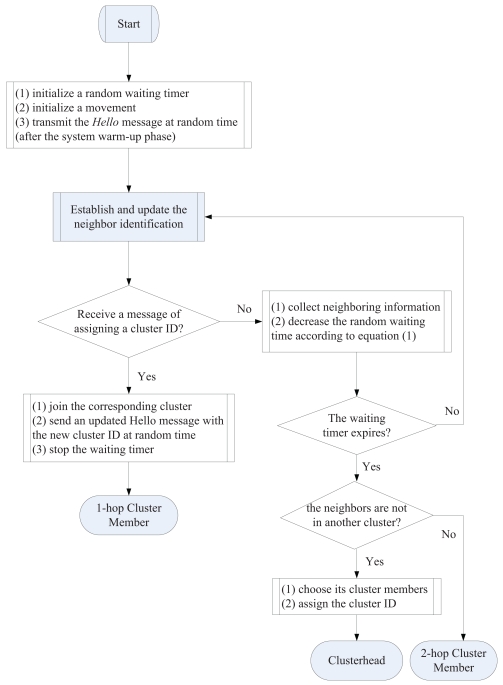
Virtual cluster construction flowchart for the SAMCA algorithm.

Given the implementation of the SAMCA, there are three different kinds of sensors: (1) the clusterheads, (2) sensors with an assigned cluster ID, and (3) sensors without an assigned cluster ID, which will join any nearby cluster and become 2-hop sensors. Thus, the topology of the ad-hoc network is now represented by a hierarchical collection of clusters. By adjusting randomized waiting timers, the sensors can coordinate themselves into sensible clusters, which can then be used as a basis for further communication and data processing. The procedures of cluster construction is outlined in the SAMCA of Figure 2.

#### Gateway Selection

Observe that the process of clusterhead selection induces non-overlapping clusters. Accordingly, to interconnect two adjacent non-overlapping clusters, one cluster member from each cluster must become a gateway This subsection presents a method of choosing distributed gateways for adjacent non-overlapping clusters. Local information, such as neighboring density and mobility of the 1-hop neighbors, is applied to select gateways and further achieve communication between clusters. The result of gateway selection is that each cluster *i* assigns a single member to communicate with each nearby cluster *j*. If the clusters are too far apart (outside the range of communication *R*), no gateway sensors will be assigned.

Denote the sensor which can communicate with more than one cluster as a border sensor. Note that only border sensors are eligible for gateway selection. Let *s_i_* be the border sensor *s* in cluster *i* and *m_j_* be the border sensor *m* in cluster *j*. *G_ij_* denotes the gateway sensor in cluster *i* that connects cluster *i* to cluster *j*. Let *d_s_i_m_j__* be the distance between sensors *s_i_* and *m_j_*, which could be estimated by the received signal strength.

Based on the cluster formation, sensors can obtain local information and know the number of neighboring sensors in adjacent clusters. When initializing the gateway selection process, clusterheads broadcast messages within the clusters. In order to achieve inter-cluster communication, a pair of border sensors with a shorter distance and similar speed may have a larger chance to be gateways. Thus, the border sensors exchange information (e.g., the distances between the neighboring sensors and their speed information) to compute the weighting values $w_{\text{ij}}^{\left(s_{i}m_{j}\right)}$, which yields

(2) $$w_{\text{ij}}^{\left(s_{i}m_{j}\right)}=c_{1}\cdot d_{s_{i}m_{j}}+c_{2}\cdot \left(\left(\upsilon_{s_{i}}+\upsilon_{m_{j}}\right)\cdot 1_{\left\{\left|\upsilon_{s_{i}}-\upsilon_{m_{j}}\right|\leq \upsilon_{T}\right\}}+2\left(\upsilon_{s_{i}}+\upsilon_{m_{j}}\right)\cdot 1_{\left\{\left|\upsilon_{s_{i}}-\upsilon_{m_{j}}\right|>\upsilon_{T}\right\}}\right),$$

where *c*_1_ and *c*_2_ are positive constants; the threshold *υ_T_* is set to be 1/2 · *υ_max_*. Given the weighting values, the operation may be divided into two stages: Contention Stage and Activation Stage. In the contention stage, each border sensor broadcasts a 1-hop message including its weighting values and the corresponding nominated pairs of distributed gateways for the nearby clusters. After a short time interval *τ_g_*, the border sensor with the minimum weighting value announces itself as the winner for the gateway selection with respect to a specific cluster and further determine the corresponding gateway for the adjacent cluster. That is,

(3) $$G_{\text{ij}}=\arg\underset{s_{i},m_{j}}{\min}\;\{w_{\text{ij}}^{\left(s_{i}m_{j}\right)}\},$$

where $\left\{w_{\text{ij}}^{\left(s_{i}m_{j}\right)}\right\}$ denotes the set of weighting measures in cluster *i* for selecting a gateway to cluster *j*. Therefore, in the activation stage, the gateway nodes, *G_ij_* and *G_ji_*, broadcast 1-hop messages to update the connectivity information and activate the linked cluster architecture.

**Figure 3.**
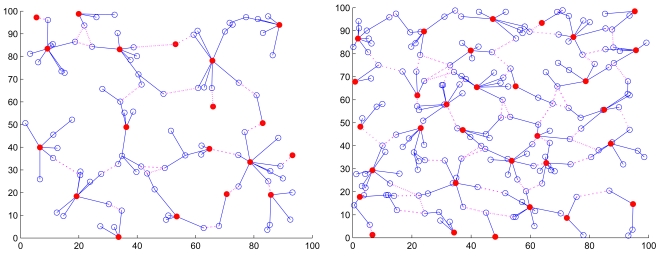
The initial cluster formation of random networks of 100 (left) and 200 (right) sensors with *R/ℓ* = 0.15 and *R/ℓ* = 0.105, respectively. A connection between a pair of distributed gateways is indicated by a dashed line.

With the node mobility *υ* = 1 m/sec, Figure 3 shows the typical runs of cluster formation of 100 and 200 sensors with *R/ℓ* = 0.145 and *R/ℓ* = 0.115, respectively, where *R/ℓ* is the ratio of transmitting range *R* to the side length of the square *ℓ* = 100 m.

### 3.2. Phase II: Cluster Reformation

The proposed cluster reformation strategy considers several cases (e.g., a sensor joining/leaving a cluster, cluster merging/splitting, and clusterhead/gateway reselection) and maintains clusters in a fully distributed way. In order to keep the hierarchical structure efficiently, load for each cluster should be equivalent. Thus, the cluster size is a key parameter to achieve balanced load among clusters. However, due to node movement, the cluster size changes as time proceeds. In the proposed approach, referring to the neighboring and its cluster sizes, each clusterhead triggers the cluster merge/split process if necessary. Let the upper bound *UB* and the lower bound *LB* represent the constraints of the cluster size for managing the topology change. When the cluster size is over the upper bound *UB*, the cluster is divided into smaller ones, while when the cluster size is under the lower bound *LB*, two neighboring clusters are merged into one cluster. Thus, each cluster size is adjusted autonomously.

Moreover, in sensor network applications, it is possible that the energy level in the clusterhead is below the threshold or the clusterhead may malfunction during the network operation. As a result, reselecting a new clusterhead may be required. For maintaining the network connectivity, gateway reselection may be necessary as well in many situations such as discovering new clusters, the link down events of the distributed gateways, merging and splitting clusters, the energy issues and so on. Therefore, this self-adaptive organization is essential in mobile wireless sensor networking systems.

#### Joining/Leaving A Cluster

When a sensor node wants to join a cluster, it may check all the links with its neighbors and measure the distances by the received signal strength. Then it chooses the nearest neighbor and joins its cluster when the cluster size constraint is satisfied. Otherwise, the sensor forms a cluster of its own, which may be merged by nearby clusters later. When a sensor leaves a cluster, this link down event can be detected by not receiving periodical broadcasting messages and the neighboring sensors can update the knowledge of its neighborhood. Other possible schemes for handling new admissions and releases of sensors in a cluster can be found in [23] and [24].

#### Merging Clusters

This subsection describes how the two neighboring clusters *C_i_* and *C_j_* combine together to form a new merged cluster. For any cluster *C_i_*, denote a set of clusters which is neighboring to cluster *C_i_* as $I_{\text{nb}}^{\left(C_{i}\right)}$ and let *N_C_i__* be the number of sensors in cluster *C_i_*. When *N_C_i__* < *LB*, clusterhead *ch_i_* determines cluster *C_j_* with the smallest cluster size as the candidate cluster for merging, where $C_{j}\in I_{\text{nb}}^{\left(C_{i}\right)}$. After determining *C_j_* as the partner of merger, clusterhead *ch_i_* sends a message to clusterhead *ch_j_* to start the merge process. In order to update the cluster ID, the clusterhead in the merged cluster may be determined by the following ways: (1) Based on the cluster size. That is, *ch_i_* is the clusterhead in the merged cluster if *N_C_i__* > *N_C_j__*; otherwise, *ch_j_* becomes the clusterhead in the merged cluster; (2) Based on the cluster ID. For instance, as *N_C_i__* = *N_C_j__* the clusterhead with a higher cluster ID is the clusterhead in the merged cluster. If *ch_i_* is the clusterhead of the merged cluster, *ch_j_* will broadcast a cluster ID update message to its cluster members in *C_j_*. Therefore, all cluster members are with the same cluster ID in the merged cluster and the merge operation is complete.

#### Splitting Clusters

**Figure 4.**
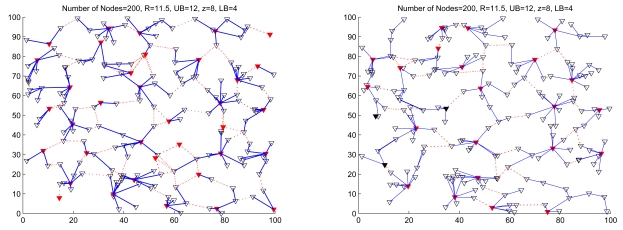
Example of cluster merge process of a random network; the network topology before cluster merging (left) and the network topology after cluster merging (right). The red ∇s represent clusterheads and the black ∇s (right) represent role changes from a clusterhead to a cluster member in the merged cluster.

**Figure 5.**
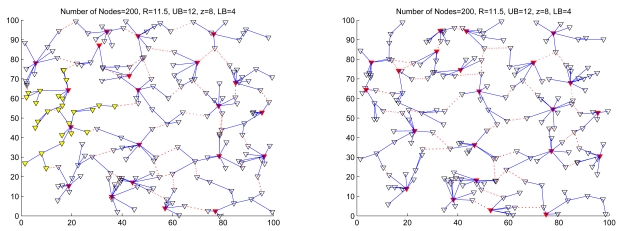
Example of cluster split process of a random network; the network topology before cluster splitting (left) and the network topology after cluster splitting (right). The yellow ∇s represent the cluster members in the split cluster (left).

In order to balance the load among clusters, the cluster with a large cluster size should be divided into smaller clusters. There are many ways to solve this problem. One possible approach is to consider the split cluster as a small network and to apply the Phase I of the SAMCA algorithm (as detailed in Section 3.1.). Thus, if the cluster size *N_C_i__* > *UB*, clusterhead *ch_i_* will send a message to its cluster members and trigger the split procedure using random timers and local information. As a result, the splitted cluster *C_i_* will efficiently distribute its load to these new formed clusters and maintain the network operation. Figure 4 and Figure 5 illustrate the cluster merge and split process of random networks, respectively.

Notice that since the neighbor sensor properties depends on the type of sensor mobility occurring in the network, the upper bound *UB* and the lower bound *LB* of the cluster size are given according to the number of sensors and issues on the sensor mobility model. Moreover, these parameters have the effect on the network maintenance since they may determine the frequency of topology change in mobile networks. Instead of using arbitrary settings for these constraint parameters [38], the analysis of the upper and lower bounds is detailed in Section 4.1.

#### Clusterhead Reselection

This subsection presents a method of choosing a new clusterhead for an existing cluster. The proposed distributed technique operates much like the SAMCA in utilizing a random timer. Once the energy in the current clusterhead is below the threshold of the energy level *ε*, it transmits a message to start the reselection process. Only those sensors with energy larger than the energy threshold *ε* are eligible. As long as the cluster member satisfies the constraint, it generates a random waiting time:

(4) $$WT_{i}^{\left(k+1\right)}=\left(1-\frac{E_{i}^{\left(k\right)}}{E_{i}^{\max}}\right)\cdot {\text{log}}_{2}\;\left(1+\frac{\left|\upsilon_{i}^{\left(k\right)}-\frac{1}{2}\left(\upsilon_{\max}+\upsilon_{\min}\right)\right|}{\frac{1}{2}\left(\upsilon_{\max}-\upsilon_{\min}\right)}\right)\cdot \rho \cdot WT_{i}^{\left(k\right)},$$

which depends on the number of neighboring cluster members, the node mobility, and the remaining energy level. Note that $E_{i}^{\left(k\right)}$ is the energy at time step *k* and $E_{i}^{\max}$ is the initial energy of sensor *i*.

For real applications, it is possible that the clusterhead may malfunction before broadcasting the re-selection message. One solution is that if a certain amount of time has passed with no messages from the clusterhead, then the cluster members begin their timers and apply the algorithm to re-initialize the network into new clusters and to help balance the energy burden.

#### Gateway Reselection

**Figure 6.**
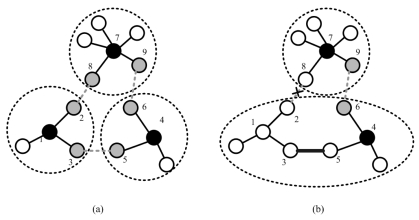
Example of distributed gateway reselection during the cluster merge procedure. The connection between a pair of distributed gateways is illustrated by a dashed line: (a) before gateway reselection and (b) after gateway reselection.

Like the clusterhead reselection, the contenders of the reselection process should satisfy the energy level constrain, which leads to the modified weighting value by multiplying a factor $\left(1-E_{i}^{\left(k\right)}/E_{i}^{\max}\right)$ to (2). When finding new neighboring clusters or splitting clusters, the corresponding border sensors may apply the gateway selection method (detailed in Section 3.1.) to establish the communication linkage. When merging two clusters, the distributed gateways of the merged cluster broadcast cluster ID update messages to inform the neighboring clusters about the topology change and further update the gateway sensors as demonstrated in Figure 6. Moreover, when the gateway sensors detect the link down events between the corresponding pairs of distributed gateways, say gateway *G_ij_* and gateway *G_ji_* for cluster *i* and cluster *j*, the gateway sensors may send a message to initialize the reselection process.

## 4. Performance Analysis

To simply the performance analysis, the mobile sensors are supposed to be uniformly distributed within the restricted region and the sensor velocity distribution is the same across the region (i.e., the random walk mobility model). By analyzing the proposed clustering scheme in different aspects qualitatively or quantitatively such as neighboring node properties, node density change, and simplified models for describing the process of cluster formation, its robustness and scalability can be clearly specified.

### 4.1. Analysis of Neighboring Sensor Properties

The sensor distribution and neighboring sensor properties in mobile ad-hoc networks are crucial metrics for describing the network performance such as connectivity and topology management. This subsection derives the statistic properties of the critical neighbor number (CNN) in a typical network topology and proposes a sensible way to select the key parameters for the SAMCA algorithm such as the *UB*, *LB*, and the reference cluster size. The following theorem investigates the condition and convergence of node distribution with respect to the mobility models.

#### 

##### Theorem 1

If the sensor velocities are independent of time and the sensor positions, the mobile sensors will asymptotically uniformly distribute over the whole given region.

According to the random walk mobility model and Theorem 1 [48], the steady-state sensor distribution should be able to maintain the uniform distribution property. Therefore, under this circumstances, the critical neighbor number may be determined by

(5) $$\text{CNN}≃E\left[N_{i}\right]=\frac{\pi R^{2}}{A}\cdot \left(n-1\right),$$

where *n* is the number of sensors, *A* is the bounded region *A* = [0, *ℓ*], *ℓ* is the side length of a square, and *R* is the transmission range of a sensor. Thus, for the network operation, the selection of the reference cluster size *z* is suggested to be

(6) $$z=\alpha \left(\text{CNN}+1\right)≃\alpha \left(E\left[N_{i}\right]+1\right),$$

where 1 ≤ *α* since the CNN may be considered as the possible number of cluster members in a cluster. In order to reduce the frequency of topology change (i.e., merging and splitting clusters), appropriate upper and lower bounds of the cluster size are necessary. Hence, *UB* may be defined by

(7) $$\text{UB}=\beta \left(\text{CNN}+1\right)≃\beta \left(E\left[N_{i}\right]+1\right),$$

where *α* < *β*. Similarly, *LB* may be expressed by

(8) $$\text{LB}=\gamma \left(\text{CNN}+1\right)≃\gamma \left(E\left[N_{i}\right]+1\right),$$

where 0 < *γ* < *α*.

Observe that the above analysis is suitable for any transmitting range. However, overly small transmission ranges may result in isolated clusters whereas overly large transmission ranges may result in a single cluster. Therefore, in order to optimize energy consumption and encourage linking between clusters, it is sensible to consider the critical transmission power which will result in a reasonably connected network. This *range assignment* problem is investigated in [9], which proves that every bounded and obstacle-free type of node mobility is detrimental for network connectivity Assume that the sensors are distributed in *A* with probability density function *ℱ_ℳ_* where *ℱ_ℳ_* is the asymptotic sensor spatial distribution based on the mobility model *ℳ* with initial deployment *ℱ*. The result is represented as the following theorem.

##### Theorem 2

*Let ℳ be an arbitrary mobility model that is bounded within A =* [0, ℓ]^2^ *and obstacle-free. Furthermore, assume that the asymptotic sensor spatial distribution ℱ_ℳ_ is continuous on the boundary ∂A, and min_A_ ℱ_ℳ_* > 0. *The critical transmission range of an ad-hoc network with the mobility model ℳ is*

(9) $$R_{ℳ}=c\cdot \ell \sqrt{\frac{\text{log}\;n}{\pi n}}$$

*for constant c* ≥ 1.

Given the random walk mobility model, Theorem 2 may suggest a good initial value for the search of optimized range assignment strategies to provide a high probability of connectivity The performance of the SAMCA with different selections of *R_ℳ_*, the reference cluster size *z*, and possible values of *UB* and *LB* (i.e the values of *α*, *β*, and *γ*), is examined via simulation.

### 4.2. Analysis of Node Density Change

This subsection analyzes the change of node density caused by the sensor movement and examines how mobility affects a cluster-based infrastructure. Since a high density distribution change means an unstable network infrastructure, the rate of the density change may be considered as an indicator of topology change. The following analysis investigates the characteristics of the link available time distribution between the clusterhead and a cluster member. Furthermore, the Lindeberg theorem [49] is applied to abstract the node density dynamics in a cluster and to examine the behavior of network operation.

#### Link Available Time

The link available time distribution of a one-hop connectivity between two sensors is described to investigate the property of the node density change rate in a cluster. At a given time *t*, consider two mobile sensors, a clusterhead and a cluster member, with the velocity vectors **v**_1_ and **v**_2_, respectively, which implies that the relative velocity vector is **v***_r_* = **v**_1_ − **v**_2_. Assume the initial distance between the clusterhead and a cluster member is *r*. Thus, the cluster member moves at a relative velocity *υ_r_* from the viewpoint of the clusterhead. After time Δ*t*, a cluster member may move out of the transmission range of the clusterhead. Consider the one-hop link between a clusterhead and a cluster member with an angle *ψ* between the line connecting these two sensor nodes and the X-axis. Suppose a cluster member moves the distance *μ* straightly within this period. Notice that given the transmission range with the mobility model *R_ℳ_*, *μ* is a function of the initial distance *r* between the clusterhead and a cluster member and the angle *θ_r_* between the relative moving direction of the cluster member and the line connecting these two sensors, *μ*(*r*, *θ_r_*). On the basis of the above settings and referring to the derivation in [50], the probability that the link available time is less than Δ*t* is given by

(10) $$P_{\text{lat}}\left(r,\psi ;\Delta t\right)=\int_{0}^{2\pi }\int_{\upsilon_{\mu }}^{\infty }f_{\upsilon_{r}}\left(\upsilon_{r},\theta_{r}\right)d\upsilon_{r}d\theta_{r},$$

where *υ_μ_* = *μ* (*r*, *θ_r_*)*/*Δ*t* and *f_υ_*(*υ_r_*, *θ_r_*) is the polar form of the relative velocity probability density function.

#### The Lindeberg Theorem

This subsection reviews the probability for analyzing the performance of the proposed scheme. Readers may refer [49] for a complete discussion and proof of the theorem.

Suppose for each *m*

(11) $$\begin{array}{c} \left(X_{11},X_{12},\ldots ,X_{1r_{1}}\right)\\ \left(X_{21},X_{22},\ldots ,X_{2r_{2}}\right)\\ \vdots \\ \left(X_{m1},X_{m2},\ldots ,X_{mr_{m}}\right) \end{array}$$

are independent random vectors. The probability space may change with *m* and (11) is called a *Triangular Array* of random variables. In the network application, denote *m* as the cluster ID information and *r_m_* represents the number of cluster members in cluster *m*. Put *S_m_* = X_*m*1_ + ⋯ + *X_mr_m__* and let *X_mi_* take the values 1 and 0 with probability *p_mi_* and *q_mi_* = 1 − *p_mi_*, respectively where *i* ∈ {1, 2, …, *r_m_*}. Thus, we may interpret *X_mi_* as an indicator that the link between clusterhead *CH_m_* and cluster member *CM_mi_* is down after time Δ*t* with probability *p_mi_* and *S_m_* is the number of link down events in cluster *m*.

Denote *Y_mi_* = *X_mi_* − *p_mi_*. Hence,

(12) $$S_{m}^{Y}\equiv \sum_{i=1}^{r_{m}}Y_{\text{mi}}=\sum_{i=1}^{r_{m}}X_{i}-\sum_{i=1}^{r_{m}}p_{\text{mi}}=S_{m}-\sum_{i=1}^{r_{m}}p_{\text{mi}},$$

(13) $$E\left[Y_{\text{mi}}\right]=E\left[X_{\text{mi}}\right]-p_{\text{mi}}=0,$$

(14) $$\sigma_{Y_{\text{mi}}}^{2}=\sigma_{X_{\text{mi}}}^{2}=p_{\text{mi}}\left(1-p_{\text{mi}}\right),$$

(15) $$\sigma_{S_{m}}^{2}=\sum_{i=1}^{r_{m}}\sigma_{Y_{\text{mi}}}^{2}=\sum_{i=1}^{r_{m}}\sigma_{X_{\text{mi}}}^{2}=\sum_{i=1}^{r_{m}}p_{\text{mi}}(1-p_{\text{mi}}).$$

For our case, the Lindeberg condition [49] reduces to

(16) $$\underset{r_{m}\to \infty }{\lim}\sum_{i=1}^{r_{m}}\frac{1}{\sigma_{S_{m}}^{2}}\int_{\left|Y_{\text{mi}}\right|\geq ɛ\sigma_{S_{m}}}Y_{\text{mi}}^{2}dP\leq \underset{r_{m}\to \infty }{\lim}\sum_{i=1}^{r_{m}}\frac{1}{\sigma_{S_{m}}^{2}}\int_{\left|Y_{\text{mi}}\right|\geq ɛ\sigma_{S_{m}}}\text{dP}=0,$$

which holds because all the random variables are bounded by 1 and [|*Y_mi_*| ≥ *εσ_S_m__*] → 0 as *r_m_* → ∞.

##### Theorem 3

*Suppose that Y_mi_ is an independent sequence of random variables and satisfies E*[*Y_mi_*] = 0, $\sigma_{Y_{\text{mi}}}^{2}=E\left[Y_{\text{mi}}^{2}\right]$, $S_{m}^{Y}=\sum_{i=1}^{r_{m}}Y_{\text{mi}}$, *and* $\sigma_{S_{m}}^{2}=\sum_{i=1}^{r_{m}}\sigma_{Y_{\text{mi}}}^{2}$. *If the Lindeberg condition (16) holds, then* $S_{m}^{Y}/\sigma_{S_{m}}\to \text{N}(0,1)$.

By Theorem 3, the distribution of the number of sensors moving out of the cluster after time Δ*t* can be approximated by $\text{N}\left(\mu_{S_{m}},\sigma_{S_{m}}^{2}\right)$ with $\mu_{S_{m}}=\sum_{i=1}^{r_{m}}p_{\text{mi}}$ and $\sigma_{S_{m}}^{2}=\sum_{i=1}^{r_{m}}p_{\text{mi}}\left(1-p_{\text{mi}}\right)$.

#### Analysis of Density Change

To proceed the investigation, let *f_D_m__*(*t*) be the node density in cluster *m* and $N_{\text{in}}^{\left(m\right)}(t)$ and $N_{\text{out}}^{\left(m\right)}(t)$ represent the number of sensors moving in and out of cluster *m* at time *t*, respectively. Therefore, at time *t* + Δ*t*, in cluster *m* the node density change Δ*f_D_m__* (*t* + Δ*t*) can be expressed by

(17) $$\Delta f_{D_{m}}\left(t+\Delta t\right)=\frac{N_{\text{in}}^{\left(m\right)}\left(t+\Delta t\right)-N_{\text{out}}^{\left(m\right)}\left(t+\Delta t\right)}{A_{m}},$$

where *A_m_* is the area size of cluster *m*. According to the criterions for merging and splitting clusters, the relationships between the node density change and the operation of cluster reformation are:

(18) $$\text{Merging}:\;\int_{t_{0}}^{t_{1}}\Delta f_{D_{m}}(\tau )d\tau <\frac{\text{LB}-N_{\text{CM}}^{\left(m\right)}(t_{0})}{A_{m}}$$

(19) $$\text{Splitting}:\;\int_{t_{0}}^{t_{1}}\Delta f_{D_{m}}(\tau )d\tau >\frac{\text{UB}-N_{\text{CM}}^{\left(m\right)}(t_{0})}{A_{m}}$$

where $N_{\text{CM}}^{\left(m\right)}(t_{0})$ is the number of cluster members in cluster *m* at time *t*_0_ and *t*_1_ is the time stamp to trigger the process of cluster reformation (*t*_1_ > *t*_0_).

Observe that the results presented in previous subsections show that *p_mi_* is actually the link available probability *P_lat_*(*r_i_*, *ψ_i_*; Δ*t*) of sensor *i* as described in (10). Since it is complex to compute the link available distribution for each cluster member and to describe the link up/down dynamics in a cluster, the simplified averaged analysis is applied to describe the topology change. Considering the mean link available time distribution between two arbitrarily chosen sensors, (i.e the mean value of link available time over all the possible positions of the cluster member), we obtain

(20) $${\bar{P}}_{\text{lat}}\left(\Delta t\right)=\int_{0}^{2_{\pi }}\int_{0}^{R_{ℳ}}P_{\text{lat}}\left(r,\psi ;\Delta t\right)\text{rdrd}\psi ,$$

which is the averaged probability that the link is available for less than Δ*t* seconds. Referring to Theorem 3, the expectation and variance of the number of link down events $N_{\text{out}}^{\left(m\right)}\left(t+\Delta t\right)$ in cluster *m* after Δ*t* seconds are

(21) $$E\left[N_{\text{out}}^{\left(m\right)}\left(t+\Delta t\right)\right]=E\left[S_{m}\right]=\sum_{i=1}^{r_{m}}p_{\text{mi}}=r_{m}{\bar{P}}_{\text{lat}}\left(\Delta t\right),$$

(22) $$\sigma_{N_{\text{out}}^{\left(m\right)}\left(t+\Delta t\right)}^{2}=\sigma_{S_{m}}^{2}=\sum_{i=1}^{r_{m}}\sigma_{X_{\text{mi}}}^{2}=r_{m}{\bar{P}}_{\text{lat}}\left(\Delta t\right)\left(1-{\bar{P}}_{\text{lat}}\left(\Delta t\right)\right).$$

Based on Theorem 1, after some time *T* the long-run sensor spatial distribution converges and the network topology is eventually stable, which suggests that for *t* > *T*, given the steady-state uniform sensor distribution, the expectation of the number of link up events $N_{\text{in}}^{\left(m\right)}\left(t+\Delta t\right)$ in cluster *m* at time *t* + Δ*t* yields

(23) $$E\left[N_{\text{in}}^{\left(m\right)}\left(t+\Delta t\right)\right]≃E\left[N_{\text{out}}^{\left(m\right)}\left(t+\Delta t\right)\right],$$

which implies that

(24) $$E\left[\Delta f_{D_{m}}\left(t+\Delta t\right)\right]=\frac{E\left[N_{\text{in}}^{\left(m\right)}\left(t+\Delta t\right)\right]-E\left[N_{\text{out}}^{\left(m\right)}\left(t+\Delta t\right)\right]}{A_{m}}≃0\;\text{for}\;t>T.$$

The frequency of topology change and the link up/down dynamics is further discussed and illustrated via simulation in Section 6.

### 4.3. Simplified Methods of Cluster Formation

Since the connectivity among sensors and the mobility of sensors play important roles in the SAMCA, it is reasonable to investigate the performance from the perspective of these parameters. Moreover, in order to understand the transition of network operation, the effect of system warm-up time on cluster formation is examined. Therefore, we abstract the behavior of the proposed algorithm in the transitional phase and steady phase of the sensor spatial distribution using two simplified models which approximate the desired global behavior and serve to analyze its performance.

#### The Transitional Phase

The first simplified model, the Mobility and Density Model (MDM), is applied to describe the behavior of the algorithm in the transitional phase without experiencing the warm-up operation. As detailed in Table 1, the basic idea of MDM is to suppose that the probability of each sensor of being a clusterhead, *p_i_*, is proportional to the number of the neighboring sensors *N_i_* and the mobility deviation during the initial deployment. That is,

(25) $$p_{i}\propto \frac{N_{i}}{\sum_{i=1}^{n}N_{i}}\cdot \left[1-{\text{log}}_{2}\;\left(1+\frac{\left|\upsilon_{i}^{\left(k\right)}-\frac{1}{2}\left(\upsilon_{\max}+\upsilon_{\min}\right)\right|}{\frac{1}{2}\left(\upsilon_{\max}-\upsilon_{\min}\right)}\right)\right].$$

If the sensor is not already chosen as a clusterhead and its neighboring sensors are not already in other clusters, then the sensor with the largest *p_i_* is chosen to be a clusterhead and it assigns probability 0 to its neighbors. Moreover, if a sensor is not a member of a cluster and some of its neighbors have already become cluster members, this sensor should choose to wait and join the nearest cluster later. After normalizing the updated probability distribution of sensors, the procedure repeats until all sensors are members of a cluster. The rationale for this choice is that since the performance of the SAMCA protocol highly depends on the connectivity among sensors and the mobility of sensors, the sensors with many neighbors and modest mobility may be good candidates for initiating new clusters. Hence, the model is likely to closely approximate the behavior of the SAMCA in the transitional phase.

#### The Steady Phase

In the steady phase of network operation, since the sensor spatial distribution converges uniformly, we may describe the network topology using the Averaged Model (AM) and the statistic properties of neighboring sensor density. The AM approximates the number of neighboring sensors that will eventually join the new cluster by the expectation of the number of neighboring sensors of each sensor in the network. Thus, a simple formula for predicting the number of clusterheads is

(26) $$N_{\text{ch}}≃\frac{n}{1+E\left[N_{i}\right]}.$$

Based on the reference cluster size *z*, upper bound *UB*, and the lower bound *LB* of the cluster size, the number of clusters *N_ch_* is bounded by the following inequality, which yields

(27) $$\frac{n}{\text{UB}}\leq N_{\text{ch}}≃\frac{n}{z}=\frac{n}{\alpha \left(1+\frac{\pi R_{ℳ}^{2}}{A}\cdot \left(n-1\right)\right)}\leq \frac{n}{\text{LB}},$$

where *α* ≥ 1.

Given the estimated number of clusters *N_ch_* and due to the close relationship between the cluster formation and gateway selection, each cluster may be symbolized as a sensor and utilize the neighboring information in the new formed network in order to determine the number of gateways in a cluster. Hence, a random network of *N_ch_* sensors with transmission range $R_{ℳ}^{2}\cdot \left(\pi N_{\text{ch}}\right)\approx c^{2}\cdot \ell^{2}\cdot \text{log}\left(N_{\text{ch}}\right)$ described in (9) may be generated to abstract the behavior of gateway selection and may approximate the number of distributed gateways in the network. Therefore, the average number of gateways in a cluster is

(28) $$N_{g(\text{avg})}=\frac{1}{N_{\text{ch}}}\sum_{i=1}^{N_{\text{ch}}}N_{i}^{\left(g\right)}≃\frac{\left(\left\lceil E\left[N_{i}\right]\right\rceil +1\right)}{n}\sum_{i=1}^{N_{\text{ch}}}N_{i}^{\left(g\right)},$$

where $N_{i}^{\left(g\right)}$ is the number of the neighboring sensors of sensor *i* in a random network of *N_ch_* sensors. The comparison of the performance of the SAMCA and the simplified models is shown experimentally in Section 6.

## 5. Energy Consumption and Complexity Analysis

This section considers the energy consumption of the SAMCA assuming homogenous sensors. The total power requirements include the power required to transmit messages *E_T_*, the power required to receive *E_R_*, and the power for processing messages *E_P_*. Moreover, the communication and time complexity are derived to assess the performance of the proposed scheme.

### 5.1. Cluster Formation

#### Clusterhead Selection

In the initialization phase, each sensor broadcasts a *Hello* message to its neighboring sensors. Therefore, the number of transmissions *N_T_x__* is equal to the number of sensors in the network, *n*, and the number of receptions *N_R_x__* is the sum of the number of neighboring sensors of each sensor. That is, *N_T_x__* = *n* and $N_{R_{x}}=\sum_{j=1}^{n}N_{j}$. As a sensor, say sensor *i*, meets the conditions of being a clusterhead, it broadcasts this and assigns cluster ID *i* to its neighboring sensors. Its neighboring sensors then transmit a signal to their neighbors to update cluster ID information. During this clustering phase, (1 + *N_i_*) transmissions and (*N_i_* + Σ*_j_*_∈_*_NB_i__N_j_*) receptions are executed, where *NB_i_* is the index set of neighboring sensors of sensor *i*. This procedure is applied to all clusterheads and their cluster members. Now let $N_{T_{x}}^{c}$ and $N_{R_{x}}^{c}$ denote the number of transmissions and receptions for all clusters, respectively. Hence, $N_{T_{x}}^{c}=\sum_{i\in I}\left(1+N_{i}\right)$ and $N_{R_{x}}^{c}=\sum_{i\in I}\left(\sum_{j\in NB_{i}}N_{j}+N_{i}\right)$, where *I* is a index set of clusterheads. Therefore, the total energy consumption, *E_cluster_*, for cluster formation in the mobile wireless sensor network is *E_cluster_* = *N_T_* · *E_T_* + *N_R_* · *E_R_*, where

(29) $$N_{T}=N_{T_{x}}+N_{T_{x}}^{c}=n+\sum_{i\in I}\left(1+N_{i}\right),$$

(30) $$N_{R}=N_{R_{x}}+N_{R_{x}}^{c}=\sum_{j=1}^{n}N_{j}+\sum_{i\in I}\left(\sum_{j\in NB_{i}}N_{j}+N_{i}\right).$$

#### Gateway Selection

The energy consumption for determining gateways is evaluated based on the description in Section 3.1. There are three possible determinations of a gateway in a cluster: (a) a 1-hop cluster member, (b) a 1-hop cluster member with a 2-hop member, and (3) a 2-hop cluster member. In order to simplify the presentation, the main notations are introduced as follows: let *I* denote the index set of clusterheads; let *H* denote the index set of 1-hop cluster members in the network; let $H_{i}^{\left(1\right)}$ denote the index set of 1-hop cluster members of cluster *i* without 2-hop neighboring cluster members (a subset of *H*); let $H_{i}^{\left(2\right)}$ denote the index set of 1-hop cluster members of cluster *i* with 2-hop neighboring cluster members (a subset of *H*); let *M* denote the index set of 2-hop cluster members in the network; let *M_i_* denote the index set of 2-hop cluster members of cluster *i* (a subset of *M*); let *G* be the index set of gateway nodes; let *B* be the index set of border nodes.

When clusterheads broadcast messages to trigger the gateway selection procedure, the number of transmission *N*_*T*_1__ and reception *N*_*R*_1__ can be expressed by

(31) $$N_{T_{1}}=\left|I\right|+\left|H_{i}^{\left(2\right)}\right|$$

(32) $$N_{R_{1}}=\sum_{i\in I}N_{i}+\sum_{i\in I}\sum_{j\in H_{i}^{\left(2\right)}}N_{j}.$$

When applying the procedure for choosing gateways, the border sensors compute the weighting values and then broadcast messages to update the connectivity information and further activate the linked cluster architecture. Accordingly, the number of transmission *N*_*T*_2__, reception *N*_*R*_2__, and processing *N_P_g__*, are given by

(33) $$N_{T_{2}}=\left|B\right|+\left|G\right|$$

(34) $$N_{R_{2}}=\sum_{j\in B}N_{j}+\sum_{i\in I}\sum_{j\in G\cap M_{i}}N_{j}+\sum_{i\in I}\sum_{j\in G\cap H_{i}^{\left(1\right)}}N_{j}+\sum_{i\in I}\sum_{j\in G\cap H_{i}^{\left(2\right)}}N_{j}.$$

(35) $$N_{P_{g}}=\left|B\right|.$$

Thus, based on the energy needed to transmit and receive, the total energy consumption for gateway selection can be assessed by *E_g_* = (*N*_*T*_1__ + *N*_*T*_2__) · *E_T_* + (*N*_*R*_1__ + *N*_*R*_2__) · *E_R_* + *N_P_g__* · *E_P_*.

#### Complexity Analysis

For clusterhead selection process, each sensor initiates 2 rounds of local flooding to its 1-hop neighboring sensors, one for broadcasting sensor ID and the other for broadcasting cluster ID, to select clusterheads and form 2-hop clusters. Hence, the time complexity is 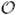
(2) rounds. For gateway selection process, the clusterhead initiates the selection operation and 2 rounds of local flooding are applied in the 2-hop cluster structure. Next, the border sensors and gateway sensors generate another 2 rounds of local flooding for broadcasting the weighting values and gateway information, respectively. Thus, the time complexity is 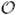
(4) rounds. Given the energy consumption analysis above, both the clusterhead selection process and the gateway selection process have a communication complexity of 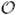
(*n*). Therefore, the communication complexity due to cluster formation is 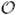
(*n*) + 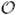
(*n*) = 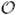
(*n*).

### 5.2. Cluster Reformation

#### Cluster Merging/Splitting

When merging two nearby clusters according to the procedures in Section , the energy consumption for determining the candidate cluster and initializing the merge process yields

(36) $$E_{m}^{\left(\text{ini}\right)}=1\cdot E_{P}+E_{T}^{\left(\text{ini}\right)}+E_{R}^{\left(\text{ini}\right)},$$

where

(37) $$E_{T}^{\left(\text{ini}\right)}≃E\left[N_{\text{hop}}^{\left(ch_{i},ch_{j}\right)}\right]\cdot E_{T}$$

(38) $$E_{R}^{\left(\text{ini}\right)}≃E\left[N_{\text{hop}}^{\left(ch_{i},ch_{j}\right)}\right]\cdot E\left[N_{i}\right]\cdot E_{R}.$$

Note that $E\left[N_{\text{hop}}^{\left(ch_{i},ch_{j}\right)}\right]$ represents the average number of hops between the two clusterheads involved in the merge operation, *E*[*N_i_*] is the average number of neighboring sensors, and *E_P_* is for determining the cluster ID of the merged cluster. Finally, a local flooding is applied within a cluster such that the cluster ID information can be unified in the merged cluster. Therefore, when combining two neighboring clusters *C_i_* and *C_j_* to form a new merged cluster and given that the merged cluster ID is *i*, the operation of updating the cluster ID requires in cluster *C_j_*

(39) $$E_{m}^{\left(\text{id}\right)}=\left(1+\left|N_{C_{j}}\right|\right)\cdot E_{P}+(1+H_{j}^{\left(2\right)})\cdot E_{T}+(\sum_{k\in H_{j}^{\left(2\right)}}N_{j}+N_{j})\cdot E_{R},$$

where *N_C_j__* is the number of cluster members of cluster *C_j_*.

For the split procedure, the energy consumption analysis in Section 5.1. can be applied since the split cluster may be regarded as a small network and the SAMCA algorithm may be used to spread the energy usage over the splitted cluster.

#### Complexity Analysis

For cluster merging procedure, $E\left[N_{\text{hop}}^{\left(ch_{i},ch_{j}\right)}\right]$ rounds of local flooding is applied to achieve an inter-cluster communication between two clusterheads. Note that $E\left[N_{\text{hop}}^{\left(ch_{i},ch_{j}\right)}\right]$ represents the average number of hops between the two clusterheads involved in the merge operation. Then, 2 rounds local flooding are applied within a cluster to unify the cluster ID information in the merged cluster. Hence, the time complexity of is $\text{O}\left(E\left[N_{\text{hop}}^{\left(ch_{i},ch_{j}\right)}\right]+2\right)$ rounds. For cluster splitting procedure, since the split cluster may apply the SAMCA to maintain the network operation, the proposed scheme has the same time complexity as described in Section. Based on the energy consumption analysis of cluster reformation above, both the cluster merging process and the splitting process have a communication complexity of 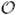
(*n*). Thus, the communication complexity due to cluster reformation is 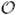
(*n*).

## 6. Simulations and Discussion

### 6.1. Experimental Settings

The scenarios are generated with input parameters such as network size, speed, transmission range, and random waiting time for clusterhead contention in order to evaluate different aspects of performance such as cluster stability, overhead consumption, network behaviors and so on.

Consider a sensor moving within the bounded region *A* = [0, *ℓ*]^2^ according to the moving pattern in [26], where *ℓ* is the side length of a square. The movement of mobile nodes is randomly generated and continuous within the whole simulation period. At some random times 0 ≤ *T*_1_ < *T*_2_ < ⋯ a new speed and a new direction are randomly selected at time *T_j_*, which initializes the *j*th movement of a sensor. Let *τ_j_* = *T_j_*_+1_ − *T_j_*, *j* ≥ 0, be the duration of the *j*th movement. During the interval [*T_j_*, *T_j_*_+1_) the sensor moves at constant speed *υ_j_* ∈ *S*, where *S* = [*υ*_min_, *υ*_max_] with 0 ≤ *υ*_min_ < *υ*_max_.

The sensor mobility is obtained by sampling from a uniform distribution with maximum value *υ*_max_ = *υ* + Δ*υ* and minimum value *υ*_min_ = *υ* − Δ*υ*, where *υ* is a given mean node speed and Δ*υ* = 1. In this work, the performance of the proposed scheme is simulated using Matlab and the experimental results are the time average over a period of 200 seconds; the duration of the *j*th movement of a sensor is *τ_j_* = 1 time unit (for ∀*j* ≥ 1); the transmission range *R_ℳ_* is determined by (9); the side length of a square is *ℓ* = 100 m.

### 6.2. The Number of Clusters

The network topology management is investigated from three perspectives: (1) with varying the *R_ℳ_/ℓ* ratio given the network density the node mobility, and the cluster formation parameters, (2) with varying the network density given the node mobility and the cluster formation parameters, and (3) with varying the degree of mobility given the network density and the cluster formation parameters.

**Figure 7.**
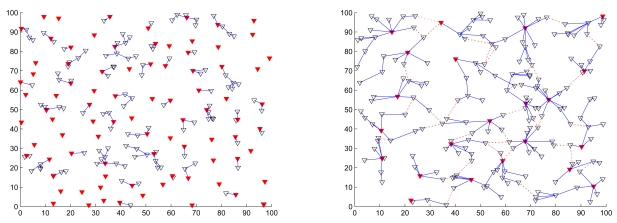
The typical runs of random networks of 200 sensors with a given mean node speed (2.5 m/sec), different *R_ℳ_/ℓ* ratios, *R_ℳ_* = 5.25 (left) and *R_ℳ_* = 10.5 (right), and the cluster parameters (*LB* = 4, *z* = 8, and *UB* = 12). Note that a red ∇ represent a clusterhead in the network and a red dashed line (right) represents a connection between a pair of distributed gateways.

From the first perspective, as shown in Figure 7, load balancing may not be achieved without an appropriate transmission range since this may lead to either too large or too small cluster sizes. Hence, the cluster formation is examined with respect to the *R_ℳ_/ℓ* ratio and network density suggested in (9) when applying the SAMCA. With random waiting time parameters *C* = 500, *λ* = 20, and *ρ* = 0.5, Figure 7 depicts typical runs of the algorithm with different *R_ℳ_/ℓ* ratios. The results show that each cluster is a collection of sensors which are up to 2 hops away from a clusterhead. Figure 7 (left) also shows that when the transmission range is small, the network with a lower sensor density will have a larger percentage of isolated sensors which eventually become clusterheads in their own right. This is because the network is only weakly connected with these values. On the other hand, given in Figure 7 (right), when the transmission power is large enough, the network is connected with high probability.

**Figure 8.**
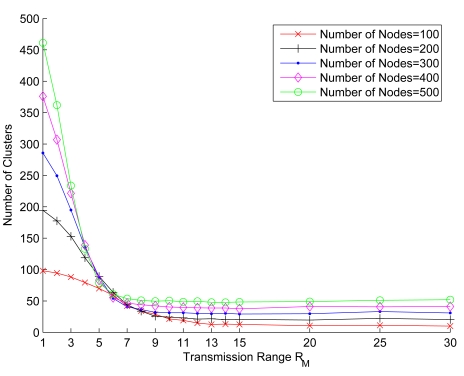
The relationship between the average number of clusters and the *R_ℳ_/ℓ* ratio with varying the number of sensors; the mean node speed = 2.5 m/sec, *LB* = 4, *z* = 8, and *UB* = 12.

Figure 8 shows the relationship between the average number of clusters and the *R_ℳ_/ℓ* ratio with varying the number of sensors. The average number of clusters in each case is the sample mean of the results of 100 typical runs. Observe that the average number of clusters decreases as the ratio *R_ℳ_/ℓ* increases (i.e., the transmission power increases). Since larger transmission power allows larger radio coverage, a cluster has more cluster members, which reduces the number of clusters in the network. However, observe that because of the cluster size constraints, the average number of clusters stabilizes as the number of sensors increases.

**Figure 9.**
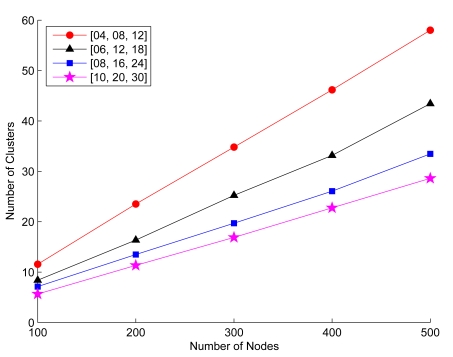
Average number of clusters for considering different cluster constraints [*LB*, *z*, *UB*] with a given mean node speed (2.5 m/sec), appropriate transmission ranges and the number of sensors; $R_{ℳ}^{\left(100\right)}=15.0$, $R_{ℳ}^{\left(200\right)}=10.5$, $R_{ℳ}^{\left(300\right)}=8.5$, $R_{ℳ}^{\left(400\right)}=7.25$, and $R_{ℳ}^{\left(500\right)}=6.25$.

From the second perspective, Figure 9 depicts that the average number of the SAMCA clusterheads increases nearly linearly with increasing network density, which demonstrates that a good selection of transmission range may lead to a minimal variation of the cluster size with increasing network density. Though the cluster maintenance operations stabilize the network topology with a variety of cluster size parameters, balancing the load among clusters with an appropriate setting for the desired number of neighbors is essential. Notice that the average cluster sizes with the parameters [*LB*, *z*, *UB*] = [4,8,12], [6,12,18], and [8,16,24] are close to the corresponding reference cluster sizes. However, the cluster formation with the parameter [10,20,30] may not spread the load among clusterheads effectively due to the wide range between the two bounds of the cluster size. The approach for determining appropriate cluster parameters is further discussed in Section 6.3.

**Figure 10.**
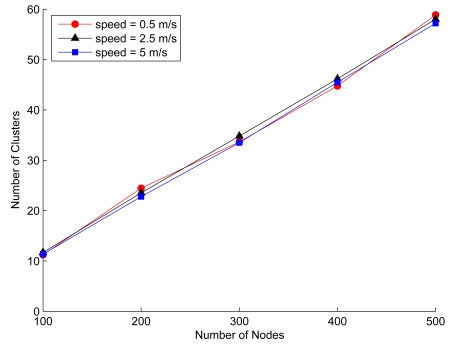
The impact of node mobility on cluster stability given the network density and the cluster formation parameters, *LB* = 4, *z* = 8, and *UB* = 12.

Considering the third perspective, Figure 10 depicts the average numbers of clusters in the network and the average cluster size for different speeds over 200 seconds, which implies that the average numbers of clusters and cluster size remain approximately stable regardless of the node moving speed. On the contrary, as increasing the node mobility, the clustering algorithms proposed in [15] and [41] organize the network with a variation in number of clusters, which may not be desirable for maintaining the network topology. Therefore, Figure 10 suggests that SAMCA may be suitable for constructing stable cluster formation in situations involving modest mobility.

### 6.3. Setting Cluster Parameters

With the random walk mobility model, the long-term sensor spatial distribution *F_ℳ_* is uniform, which suggests that the values of neighbors derived for stationary, uniformly distributed networks can be applied to characterize the reference cluster size *z*. In reality, maintaining full connectivity in a mobile network is challenging. In this work, given *α* = 1, a proper transmission range, and the network density, a possible choice for the reference cluster size as described in (6) yields

(40) $$z≃\alpha \;\left(\left(n-1\right)\cdot \frac{\pi R_{ℳ}^{2}}{\ell^{2}}+1\right)≃8,$$

which coincides with the result presented in [9] that having a number of neighbors in the range 6 ∼ 8 (i.e. a cluster size in the range 7 ∼ 9) may provide a reasonably connected communication graph in a mobile ad-hoc network.

**Figure 11.**
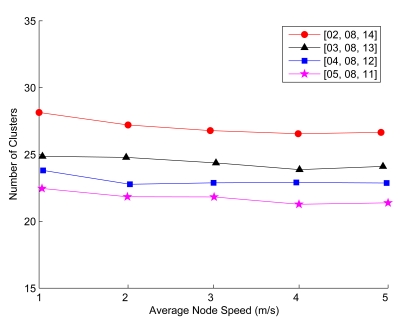
The impact of cluster parameter selection on cluster formation over a time period of 200 seconds.

Accordingly, Figure 11 illustrates the impact of cluster parameter selection on network topology by investigating the parameters *α*, *β* and *γ* for determining *UB* and *LB* of cluster size (detailed in Section 4.1.). With *α* = 1, *β* = (1 + Δ*k*)*α*, and *γ* = (1 − Δ*k*)*α*, the graph depicts the network performance for varying Δ*k*, where Δ*k* = 0.375, 0.5, 0.625, and 0.75, respectively. Note that by configuring these cluster parameters, the network topology considerations can be clearly specified. When Δ*k* is large, it may introduce a variation of the cluster size in the network. However, when Δ*k* is small, the number of clusterhead changes may increase because of the process of frequent cluster reformation. Hence, the above results allow to elucidate the global network behavior and to set the appropriate parameters for shaping and controlling the network operation.

### 6.4. The Number of Clusterhead/Gateway Changes

**Figure 12.**
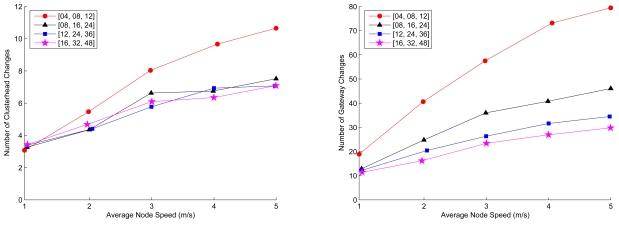
Average number of clusterhead (left) and gateway (right) changes with varying the node mobility and the cluster constraint parameters; the number of sensors *n* = 200 and *R_ℳ_* = 10.5.

Figure 12 (left) shows the number of clusterhead changes. In order to utilize the hierarchical network structure, it is preferable that the number of changes of clusterheads becomes less. For a 1-hop cluster-based topology, due to the node movement, the cluster structure may not be stable and the ripple effect may be generated under this circumstances. On the other hand, in SAMCA, as the node moving speed becomes faster, the number of clusterhead changes increases only slowly. This is attributed to the fact that the clusters are reconfigured autonomously as the topology changes.

With parameters *c*_1_ = 1 and *c*_2_ = 1 as described in (2), Figure 12 (right) illustrates the number of gateway changes. Note that the proposed method provides a way to extract the behaviors of the border sensors with modest mobility during the process of gateway selection. However, the changes in gateways increase for the sensor nodes with high mobility, which may adversely affect the performance of the clustering algorithm. Therefore, an alternative approach is that distributed gateways can be composed by a group of the border sensors and local flooding may be applied to achieve data dissemination and provide link availability between adjacent clusters in a high mobility ad-hoc sensor networks.

### 6.5. The Effect of Merging/Splitting Clusters

**Figure 13.**
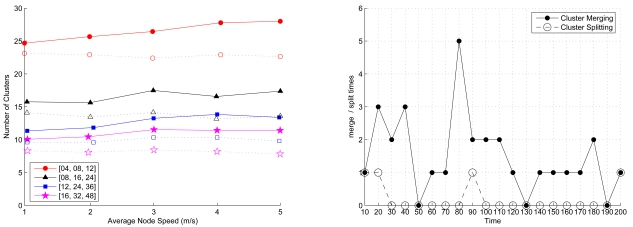
The comparison of the initial cluster formation (filled symbol) and the stable one (hollow symbol) for different settings of cluster parameters (left); the number of cluster merging and splitting in a network given the network density *n* = 200, *R_ℳ_* = 10.5, the mean node mobility = 2 m/sec, and the cluster formation parameters (*LB* = 4, *z* = 8, and *UB* = 12).

Given the network density, the node mobility, and the cluster formation parameters, Figure 13 (left) depicts the comparison of the initial cluster formation and the stable one for different settings of cluster parameters and Figure 13 (right) describes the frequency of cluster merging and splitting in a network over a time period of 200 seconds. As shown in Figure 13, the number of cluster reformation operation naturally decreases and the network topology towards to be stable as time proceeds.

**Figure 14.**
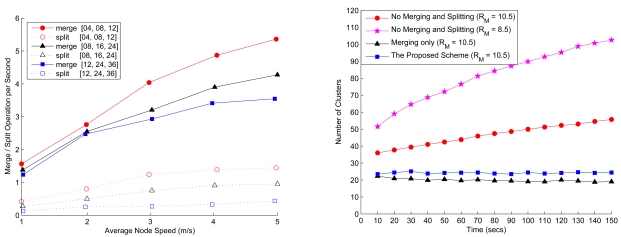
The frequency of cluster merging and splitting in a network given the network density *n* = 200 with varying the node mobility and the cluster formation parameters (left); the comparison of the network topology without cluster reformation and the network topology with cluster reformation given the network density *n* = 200, the transmission range *R_ℳ_*, the node mobility = 2 m/sec, and the cluster formation parameters (*LB* = 4, *z* = 8, and *UB* = 12) (right).

Figure 14 (left) depicts the number of clusters when merging and splitting are employed for different transmission ranges. Compared with the cluster formation using a large transmission range, a short transmission range results in a larger number of clusters since the sensors may be isolated or form clusters locally. Moreover, when the transmission range is large, the large cluster range may leads to unbalanced load among clusters. Therefore, in order to achieve load balancing in each cluster, the merge and split technique can be applied to reorganize the network. With merging and splitting, Figure 14 (right) illustrates that when compared to the network topology without cluster reformation, the topology with cluster reorganization has less clusters, which further stabilize the network operation with a small variation in the number of clusters.

**Figure 15.**
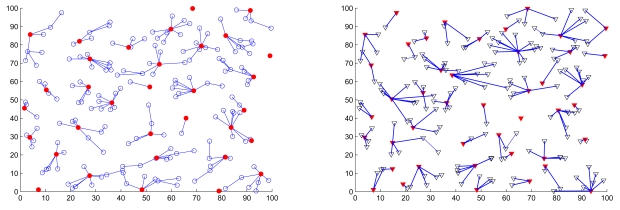
The comparison of a typical network topology applying the proposed scheme (left) and that using the MOBIC [32] algorithm (right) over a time period of 200 seconds.

Figure 15 shows the comparison of a typical network topology applying the proposed scheme (left) and that using the MOBIC algorithm (right). As mentioned in Section 2., MOBIC presents an aggregate local mobility metric for the cluster formation process such that mobile sensors with low relative speed have the chance to become clusterheads. However, it may not work well in a dynamic environment due to the disregard of mobility manners in cluster maintenance. On the other hand, the proposed approach configures the network autonomously with cluster formation parameters and considers mobility manners in cluster reformation phase when facing the topology changes, which manages the topology effectively.

### 6.6. Energy Consumption

**Figure 16.**
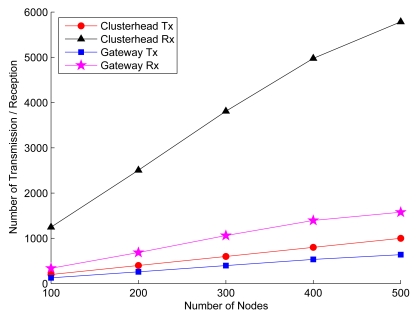
The energy consumption for cluster formation: the clusterhead selection and distributed gateway selection.

This set of experiments considers the energy consumption of cluster formation (Phase I) in the SAMCA scheme. Assume that the communication channel is error-free and the mean node mobility is *υ* = 1 m/sec. Given appropriate transmission ranges for various network densities, Figure 16 shows the average number of transmissions and receptions of random networks for selecting clusterheads and gateways, respectively As derived in Section 5., the number of receptions tends to increase as the ratio *R_ℳ_/ℓ* increases, which implies that energy consumption is higher for the network with larger transmission power. This can be attributed to the fact that larger transmission power allows sensors to detect more neighbors, which increases the number of receptions when assigning cluster ID, updating cluster ID information, or broadcasting gateway information.

Referring to the analysis in Section 5.1. and the experimental result, the proposed self-organization scheme ( 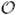
(4), 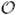
(*n*)) for cluster formation is demonstrated to be comparable to Lowest-ID algorithm ( 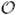
(1), 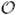
(*n*)) [21], the Max-Min heuristic ( 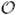
(*D*), 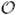
(*n*)) [16], and the tree-partitioning technique for grouping sensors ( 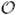
(*S*), 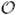
(*n*)) [51] in terms of the time complexity and the communication complexity, where *n* is the number of sensors in the network, *D* is the maximum number of hops that an ordinary node can be away from its clusterhead, and *S* is the predefined cluster size.

### 6.7. Comparison of Analysis and Simulation

#### Link Dynamics (*N_out_*)

**Figure 17.**
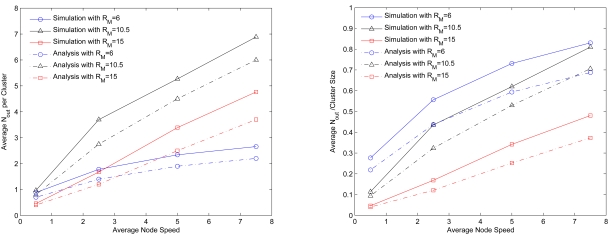
The average *N_out_* in a cluster for varying the node speed (left) and the ratio of the average *N_out_* to the average cluster size for varying speed (right); the average cluster sizes for *R_ℳ_* = 6, *R_ℳ_* = 10.5, and *R_ℳ_* = 15 are 3.18, 8.82, and 9.93, respectively.

The effect of mobility on the link down rate is studied with varying transmission ranges. The various properties investigated in Section 4. characterize the behavior of the links of mobile sensors, which can be used as a basis for analyzing the performance bounds of the proposed SAMCA protocol. Thus, the link dynamics may be used as an indicator of topology change and the distribution of link available time can be used to examine the link stability in the network. Given the network density (*n* = 200), the transmission range *R_ℳ_*, and the cluster formation parameters (*LB* = 4, *z* = 8, and *UB* = 12), Figure 17 illustrates the comparison of the theoretical analysis and simulation results for characterizing the average number of link down event *N_out_* in a cluster within Δ*t* = 1 second during the observation time 200 ∼ 201 second with varying the node speed. As expected, the average number of of link down event *N_out_* in a cluster increases with increasing the node mobility. Moreover, Figure 17 also implies that the theoretical analysis and the simulation result match well and the characteristic of the link down event can be roughly monitored and described by a function of the link lifetime distribution as detailed in Section 4.2.

#### Simulations vs. Simplified Models

**Figure 18.**
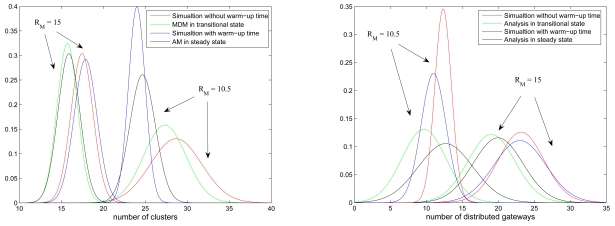
The comparison of the number of clusterheads (left) and distributed gateways (right) when applying the SAMCA approach and the simplified models.

The network behaviors in the transitional and steady phases are explored when applying the SAMCA approach and the simplified models in a random network of 100 sensors. In each method, the results of 100 typical runs are merged. Given in Figure 18 are the standard deviations of the mean number of clusterheads (left) and gateways (right) with varying the transmission power *R_ℳ_/ℓ*, which shows the comparison of the initial cluster formation (without warm-up time) and the Mobility and Density Model (MDM) in the transitional phase and the comparison of the simulated results (with warm-up time) and the Averaged Model (AM) in the steady phase.

The graph suggests that the MDM method and the AM method well approximate the SAMCA performance. This is reasonable because the MDM retains global connectivity and mobility information for cluster construction in the transitional phase. Due to the uniform convergence of the sensor spatial distribution, the approximation of the AM method may be a way to predict the performance of the SAMCA. Moreover, with an appropriate transmission range (*R_ℳ_* = 15.0), Figure 18 also shows that both the network behaviors during the transitional phase and the steady phase are close, which may imply that the effect of the system warm-up operation on the network performance is not significant since the whole network is reasonably connected. On the other hand, without a proper transmission range (*R_ℳ_* = 10.5), experiencing the system warm-up time may help stabilize the number of clusters under the control of the proposed self-organization scheme.

### 6.8. Comparison of Cluster-Based Self-Organization Schemes

The final set of experiments compares the robustness of the SAMCA algorithm with other cluster-based self-organization schemes. Simulation study is conducted to show that the performance of the ESAC [43] approach is superior to those in [3, 33, 41, 42]. In ESAC, both diameter and size of the cluster are restricted in the purpose to generate balanced clusters. Two thresholds Thresh*_Lower_* and Thresh*_Upper_* are applied to control the size of clusters. Moreover, the distance between cluster members and their corresponding cluster-head is at most two hops. Based on the above characteristics of cluster formation, the ESAC heuristic may provide a good way to benchmark the performance of SAMCA scheme. To compare SAMCA with ESAC, the cluster stability is investigated given the network density and cluster formation parameters. For the ESAC heuristic, experiments are performed with two distinct values for threshold Thresh*_Upper_* = 10 (ESAC_10) and Thresh*_Upper_* = 15 (ESAC_15) and a fixed value for threshold Thresh*_Lower_* = 5. For the SAMCA scheme, the values of cluster formation parameters are *LB* = 4, *z* = 8, and *UB* = 12 (SAMCA[4, 8, 12]).

**Figure 19.**
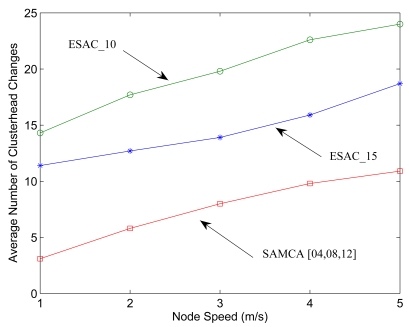
The average number of clusterhead changes using the SAMCA and the ESAC heuristic. *n* = 200, *R/l* = 0.105.

Figure 19 shows and compares the average number of clusterhead changes when applying the ESAC and the proposed SAMCA. Observe that, unlike the LCC algorithm [42], both the ESAC and SAMCA schemes ensure cluster stability when nodes' speed increases. However, the frequency of clusterhead changes in ESAC is higher than that of the SAMCA with varying the node speed. Thus, the SAMCA algorithm may be more suitable for network topology control since the frequent clusterhead changes may degrade the system performance. The above set of experiments implies that the SAMCA is competitive with the ESAC heuristic in terms of cluster formation and topology management. Furthermore, [43] shows that more study may be needed for determining appropriate times to trigger the operation of cluster maintenance in the ESAC heuristic. In comparison, the SAMCA may be reliably applied to govern the topology change.

## 7. Conclusion

This paper introduces an adaptive distributed clustering scheme for mobile wireless sensor networks. The proposed algorithm performs cluster formation and linkage using random waiting timers and local information. It investigates the key features of cluster construction and maintenance such as transmission range and constraints of cluster size so that the load and overhead of clusterheads in each cluster can be balanced. As shown in simulation experiments, the proposed scheme achieves cluster stability and adaptability in mobile sensor networking systems. Moreover, the experimental results also demonstrate that the simplified approximate models and analysis can well describe the network behaviors, which suggests that the approximation may be a sensible way to assess the performance of the proposed algorithm. On the basis of the cluster-based network topology, this self-configuring technique can be applied to provide efficient topology management in mobile wireless sensor networks.

## Figures and Tables

**Table 1.** The Mobility and Density Model.

Assign a probability to sensor *i*, *p_i_*, proportional to the number of the neighboring sensors, *N_i_* and the mobility deviation as shown in (25). Let *B_i_* be the set of neighboring sensors of sensor *i*. *I* is the index set of clusterheads. **P**^(*k*)^, **P̄**^(*k*)^, and **P̃**^(*k*)^ are 1 by *n* vectors to store the probability distribution at time step *k*. Assign *k* = 0 and **P**^(0)^ = (*p*_1_, *p*_2_, …, *p_n_*). **while** (sum(**P**^(*k*)^) > 0) Select a clusterhead $j=\arg\max_{i}\left\{p_{i}^{\left(k\right)}\right\}$, *j* ∈ *I*. Update the probability distribution ${\tilde{p}}_{i}^{\left(k\right)}=p_{i}^{\left(k\right)}\cdot 1_{\left\{i\notin B_{j},B_{i}\cap B_{j}=\emptyset ,j={\text{arg max}}_{i}\left\{p_{i}^{\left(k\right)}\right\}\right\}}$, *p̃_j_*^(*k*)^ = 0. Normalize the updated probability distribution. **if** (sum (**P̃**^(*k*)^) > 0) *p̄_i_*^(*k*)^ = *p̃_i_*^(*k*)^/sum(**P̃**^(*k*)^). **else** **P̄**^(*k*)^ = **P̃**(*k*). **end** Store the normalized probability distribution. **P**^(*k*)^ = **P̄**^(*k*)^, set *k* = *k* + 1. **end**
